# Transcriptome Profiles of the Protoscoleces of *Echinococcus granulosus* Reveal that Excretory-Secretory Products Are Essential to Metabolic Adaptation

**DOI:** 10.1371/journal.pntd.0003392

**Published:** 2014-12-11

**Authors:** Wei Pan, Yujuan Shen, Xiuming Han, Ying Wang, Hua Liu, Yanyan Jiang, Yumei Zhang, Yanjuan Wang, Yuxin Xu, Jianping Cao

**Affiliations:** 1 National Institute of Parasitic Diseases, Chinese Center for Disease Control and Prevention, Shanghai, People's Republic of China; 2 Key Laboratory of Parasite and Vector Biology, Ministry of Health, Shanghai, People's Republic of China; 3 WHO Collaborating Center for Malaria, Schistosomiasis and Filariasis, Shanghai, People's Republic of China; 4 Department of Parasitic Diseases, Qinghai Institute for Endemic Disease Prevention and Control, Zong Zhai, Xining, Qinghai, People's Republic of China; University of Queensland, Australia

## Abstract

**Background:**

Cystic hydatid disease (CHD) is caused by the larval stages of the cestode and affects humans and domestic animals worldwide. Protoscoleces (PSCs) are one component of the larval stages that can interact with both definitive and intermediate hosts. Previous genomic and transcriptomic data have provided an overall snapshot of the genomics of the growth and development of this parasite. However, our understanding of how PSCs subvert the immune response of hosts and maintains metabolic adaptation remains unclear. In this study, we used Roche 454 sequencing technology and *in silico* secretome analysis to explore the transcriptome profiles of the PSCs from *E. granulosus* and elucidate the potential functions of the excretory-secretory proteins (ESPs) released by the parasite.

**Methodology/Principal Findings:**

A large number of nonredundant sequences as unigenes were generated (26,514), of which 22,910 (86.4%) were mapped to the newly published *E. granulosus* genome and 17,705 (66.8%) were distributed within the coding sequence (CDS) regions. Of the 2,280 ESPs predicted from the transcriptome, 138 ESPs were inferred to be involved in the metabolism of carbohydrates, while 124 ESPs were inferred to be involved in the metabolism of protein. Eleven ESPs were identified as intracellular enzymes that regulate glycolysis/gluconeogenesis (GL/GN) pathways, while a further 44 antigenic proteins, 25 molecular chaperones and four proteases were highly represented. Many proteins were also found to be significantly enriched in development-related signaling pathways, such as the TGF-β receptor pathways and insulin pathways.

**Conclusions/Significance:**

This study provides valuable information on the metabolic adaptation of parasites to their hosts that can be used to aid the development of novel intervention targets for hydatid treatment and control.

## Author Summary

The successful infection establishment of parasites depends on their ability to combat their host's immune system while maintaining metabolic adaptation to their hosts. The mechanisms of these processes are not well understood. We used the protoscoleces (PSCs) of *E. granulosus* as a model system to study this complex host-parasite interaction by investigating the role of excretory-secretory proteins (ESPs) in the physiological adaptation of the parasite. Using Roche 454 sequencing technology and *in silico* secretome analysis, we predicted 2280 ESPs and analyzed their biological functions. Our analysis of the bioinformatic data suggested that ESPs are integral to the metabolism of carbohydrates and proteins within the parasite and/or hosts. We also found that ESPs are involved in mediating the immune responses of hosts and function within key development-related signaling pathways. We found 11 intracellular enzymes, 25 molecular chaperones and four proteases that were highly represented in the ESPs, in addition to 44 antigenic proteins that showed promise as candidates for vaccine or serodiagnostic development purposes. These findings provide valuable information on the mechanisms of metabolic adaptation in parasites that will aid the development of novel hydatid treatment and control targets.

## Introduction

Cystic hydatid disease (CHD) is a serious parasitic zoonosis that is caused by the larval stages of *Echinococcus granulosus*, a cestode that poses a threat to public health as well as significant economic losses [1], [2], [3]. At present, more than 3 million people are infected with this parasite [4], [5], and the prevalence reaches 10% in some areas [6], [7]. The disease is difficult to control because appropriate diagnostic procedures are lacking and the available drugs are inefficient [8].

*E. granulosus* has a complex developmental cycle, involving eggs, oncospheres, protoscoleces (PSCs), and adult stages. Adult parasites live in the small intestine of dogs. After sexual maturation, numerous eggs are produced by the adult parasites and are then excreted with the dog feces. Infections occur in an intermediate host, when eggs containing larvae are ingested. Hydatid cysts (the larval stage or metacestode) develop in the internal organs (primarily in liver and lungs) of intermediate hosts. The larval stages of *E. granulosus* are comprised of two layers of cyst wall: cyst fluid and PSCs [9].

As the only infectious form of the larval stages, PSCs can interact with both definitive and intermediate hosts. They mature into adult parasites when the hydatid cysts are ingested by the definitive host. They can also differentiate into new cysts when released into the body cavity of intermediate hosts upon cyst rupture [10]. Mouse models of CHD are often established *via* the intraperitoneal inoculation with PSCs, a method that has been widely applied to drug screening and vaccine development [11], [12]. Overall, the PSC is an important infectious reagent that contributes to the transmission of CHD and also an excellent model system in which many aspects of the host-parasite interaction can be studied.

Understanding the elaborate immune evasion strategies and mechanisms of physiological adaptation of the PSCs is critical to ascertain effective intervention targets to control the prevalence of the parasite. In this study, we focus on the role of excretory-secretory products (ESPs) that are released by parasites, as these compounds are exposed directly to the immune system of the hosts and are engaged at the host-parasite interface [13]. The mechanism by which PSCs can subvert the immune environment *via* ESPs is the key to successful infection. Recently, we found that ESPs from adult *E. granulosus* could downregulate host immune responses by preventing dendritic cells (DC) from maturing, by impairing DC function and by inducing the generation of CD4^+^ CD25^+^ FoxP3^+^ T cells (unpublished data). Previous studies have shown that cystic fluids produced in the intermediate hosts can modulate DC differentiation and cytokine secretion [14], while antigen B released by the germinal cells of *E. granulosus* can direct immature DCs towards the maturation of a Th2 cell response [15]. Moreover, the ESPs from *E. multilocularis* larvae have been found to induce apoptosis and tolerogenic properties in DC *in vitro* [16]. To date, studies have focused primarily on the immune regulation of ESPs by the host, with little work undertaken to investigate the influence of ESPs on the physiological adaptation of parasites to their hosts. Interestingly, several intracellular proteins that were not previously thought to be exposed to the immune system of hosts have recently been identified in the ESPs of PSCs [9], [17]. This finding suggests that parasite-derived ESPs are incorporated in the metabolites of the host [18], [19].

Further investigations into the mechanisms of physiological adaptation of ESPs released by PSC have been hampered due to the paucity of information regarding ESPs. Although studies have utilized proteomics to identify the constituents of ESPs [9], [20]–[22], very few have been identified. This is largely because of interference from host proteins [20]–[21] and because of technical limitations of the methodologies used. In recent years, however, the combination of transcriptomics and proteomics has enabled the identification of an increasing number of parasitic proteins [23], [24].

In this study, we used Roche 454 sequencing technology and *in silico* secretome analysis to explore the transcriptome profiles of *E. granulosus* PSCs and to elucidate the potential functions of the ESPs released by the parasite.

## Materials and Methods

### Ethics statement

This study was performed in strict accordance with the recommendations provided in the Guide for the Care and Use of Laboratory Animals of the National Institute of Parasitic Diseases, Chinese Center for Disease Control and Prevention. The protocol was approved by the Laboratory Animal Welfare & Ethics Committee (LAWEC), National Institute of Parasitic Diseases, Chinese Center for Diseases Control and Prevention (Permit Number: IPD 2011-006).

### Sample collection

Hydatid cysts were collected from the livers of a naturally infected sheep in a slaughterhouse in Qinghai, China. Cyst fluids containing PSCs were sucked out of the cysts using a sterile syringe. After natural sedimentation for 10 min, PSCs were carefully collected from the sediment of cyst fluids and washed 10 times with saline solution. We then added 2 mL of Trizol reagent (Invitrogen, USA) to the well-washed PSCs. After continuous mixing with a pipette, the PSCs were stored at −80°C prior to use.

### Genotyping the PSCs

Genomic DNA from the PSCs was extracted using the DNeasy tissue kit (Qiagen, Hilden, Germany) and used as a template for a polymerase chain reaction (PCR) [25]. The following two primer pairs were used to amplify the mitochondrial genes of *Echinococcus* species: cytochrome coxidase subunit 1 (*cox1*) gene (F: 5′-TTGAATTTGCCACGTTTGAATGC-3′; and R: 5′-GAACCTAACGACATAACATAATGA-3′) and cytochrome b (*cytb*) gene (F: 5′-GTCAGATGTCTTATTGGGCTGC-3′; R: 5′-TCTGGGTGACACCCACCTAAATA-3′). Each 25-µL reaction mixture contained 1 µL of template DNA, 12.5 µL Premix Taq® mix (TaKaRa Biomedicals, Tokyo, Japan), l µL of 10 µM of each primer, and 9.5 µL nuclease-free water. The procedure of PCR amplification consisted of 94°C for 1 min, 30 cycles of 94°C for 30 s, 56°C for 30 s, and 72°C for 1 min, followed by 72°C for 10 min, with a final holding step at 4°C. The PCR products were directly sequenced with a Dye Terminator Cycle Sequencing Kit (Amersham Biosciences, Tokyo, Japan) and ABI 3730 DNA Analyzer (Applied Biosystems, Foster City, USA).

### cDNA library preparation, Roche 454 sequencing and sequence assembly

The total RNA was extracted from the PSCs in TRIzol reagent, and RNA quality was performed by gel electrophoresis with a 2100 BioAnalyzer (Agilent Technology, Santa Clara, USA). The sequencing protocol followed that described in Liao *et al.* [26], and was carried out at the Shanghai OE Biotech Company. cDNA was synthesized using 2 µg of total RNA with the SMART cDNA synthesis kit (Clontech Laboratories, Mountain View, USA) according to the manufacturer's instructions. The cDNA library was constructed using a GS-FLX Titanium General Library Preparation Kit (Roche, Branford, USA) without normalization [27], and then sequenced using a half run on the Roche 454 GS-FLX Titanium platform. The modules built-in Newbler 2.5.3 (a *de novo* sequence assembly software, Roche, USA) was used to remove low quality sequences and assemble the remaining sequences. Briefly, the quality score trimming filter trims back from the 3′ end of reads and was based on estimated quality scores (not the final quality scores) derived from an internal calibrated signal histogram. The error rate in a sliding window (default size of 40 bp) was calculated from the estimated quality scores and multiplied by an empirical scaling factor (default of 1.1). The window was moved leftwards until the estimated error rate in the window was <1.0% (by default). If the resulting read was less than 40 bp (default), the read was discarded and not counted (numTrimmedTooShortQuality metric). After removing low quality sequences and sequencing adaptors, the remaining sequencing reads were assembled using the Newbler 2.5.3 with the ‘extend low depth overlaps’ parameter. All of the ESTs from the Roche 454 were used to run the final assembly. The resulting isotig consensus sequences and singletons were referred as ‘unigenes’ in the following study.

### Bioinformatic analyses of transcriptomic sequence data

The software SOAP2 was used to map the raw sequence reads to the nonredundant sequence data [28]. Briefly, raw reads were aligned to the assembled, nonredundant transcriptomic data, to ensure that each read was mapped to a unique transcript. Reads mapped to more than one transcript were randomly assigned to one unique transcript, to ensure that they were recorded only once. Reads per kilobase per million reads (RPKM), the evaluation index of relative assessment of transcript abundance, was calculated using the standard formula [29].

Unigene sequences were compared (using BLASTn with a cutoff E-value of 1e-5) to public sequences available in NCBI non-reductant (Nr) and STRING databases, and to five entire genome sequences (*E. multilocularis* [30], *E. granulosus* [31], *Schistosoma hematobium* [32], *S. japonicum* [33], *S. mansoni* [34]).

After conceptual translation from the predicted coding domains of individual transcriptomic sequences, the functions of the potential proteins were predicted using InterProScan [35], employing the default parameters. According to their homology with conserved domains and with protein families, proteins inferred for *E. granulosus* PSC (*Eg*PSC) were assigned to three gene ontology (GO) categories, including molecular function, cellular component and biological process [36]. The pathway analysis of inferred proteins was carried out using the KEGG (Kyoto Encyclopedia of Genes and Genomes) database [37].

### *In silico* secretome analysis

Excretory-secretory proteins (ESPs) were predicted according to the methods described by Garg and Ranganathan [38], [39]. Briefly, the secretory proteins were predicted utilizing the following five tools: ESTScan 3.0.3 [40] to translate the unigenes into putative proteins; SecretomeP 1.0 [41] for non-classical secreted proteins; SignalP 4.1 [42] for classical secreted proteins; TargetP 1.1 [43] for trimming mitochondrial proteins; and TMHMM 2.0 [44] for trimming transmembrane proteins. The predicted proteins with no transmembrane helices were thought to be ESPs.

In addition to traditional computational approaches for ESPs prediction, we also predicted *E. granulosus* ESPs (*Eg*ESPs) using BLASTP [45]. Based on their homology, a list of ESP sequences that included 478 nucleotides and 1,126 proteins was obtained to extract ESPs from the proteins that were predicted to be non-secretory by SecretomeP. Those ESPs had been identified in experiments in other species (*S. mansoni, S. japonicum, Brugia malayi, Ancylostoma caninum, Teladorsagia circumcinta, Fasciola hepatica* and *Clonorchis sinesis*) [46]–[59]. In this approach, a correct match for protein (Query) to protein (Subject) was designated when the query ratio was>80% of their length and identity ≥60, while a correct match for protein (Query) to nucleic acids (Subject) was designated when the query ratio was>80% of their length and identity ≥90.

All potential ESPs were blasted with known ESP sequences from *E. granulosus* (including nucleotide and protein sequences [9], [7], [20]–[22] and our unpublished data) to validate the *in silico* secretome analysis. They were then annotated against GO, KEGG, Reactome (http://www.reactome.org/ReactomeGWT/entrypoint.htm1) and Panther (http://www.patherdb.org/) databases to identify functional groups and pathway annotations. Enrichment of KEGG pathways for genes with significant expression was calculated utilizing a classical hypergeometric distribution statistical comparison of the query gene list against all predicted *E. granulosus* genes. *Caenorhaditis elegans* pathways were used as a reference. Calculated *P*-values were subjected to FDR correction, with *p*<0.05 taken as the threshold for significance.

### Accession number

The transcriptome data is stored in Sequence Read Archive (SRA, No. SRP040541, http://www.ncbi.nlm.nih.gov/sra/?term=SRP040541).

## Results/Discussion

### Genotyping of *E. granulosus* PSCs

The genotype of *E. granulosus* PSCs used in this study was sheep G1, as the PCR fragment amplified from *cytb* gene showed the highest identity (99%) to the *E. granulosus* G1 genotype referenced in GenBank (accession AF297617, S1 Figure). This was consistent with the fact that sheep G1 strain is the most common strain worldwide [60].

### Roche 454 transcriptome sequencing and reads assembly

A total of 330,188 raw reads (mean length = 411.8 bp) were generated. The data is stored in Sequence Read Archive (SRA, No. SRP040541). After trimming to remove adaptors, low quality reads and polyN tail sequences, 329,927 clean reads remained (mean length = 400.3 bp; Table 1). Clean reads were assembled and produced about 26,514 unigenes ranging in size form 150–3,357 bp (mean = 501.5 bp). These included 4,175 isotigs ranging in size from 154 to 3,357 bp and 22,339 singletons of 150 to 1,710 bp. Approximately 84% of the isotigs were>500 bp, while most singletons (85.97%) were between 300 and 800 bp in size (Table 1, S2 Figure). The numbers of *Eg*PSCs unigenes matching known sequences are listed in Table 1. In summary, 26,514 unigenes were inferred from our transcriptome. The large majority of these (17,861, 67.4%) exhibited the highest level of homology to proteins in *E. multilocularis*, followed by proteins from *E. granulosus* (17,732; 66.9%), *Caenorhabditis elegans* (8,946; 33.7%) and *S. mansoni* (2,159; 17.5%). Moreover, 22,910 (86.4%) contigs were mapped to the *E. granulosus* genome and 17,705 (66.8%) of these were distributed within the coding sequence (CDS) region, which suggested that our results were reliable.

**Table 1:** Summary of the nucleotide sequence data for EgPSCs prior to and following assembly, with detailed bioinformatic annotation and analyses.

Raw reads	330188
Unigenes (average length; min-max length)	26514 (510.5; 150–3357)
Containing an open reading frame (%)	19576 (73.8)
With homologues in *E. granulosus* (%)	17732 (66.9)
*E. multilocularis*	17861 (67.4)
*Caenorhabditis elegans*	8946(33.7)
*Clonorchis sinensis*	2540 (20.6)
*Schistosoma mansoni*	2159 (17.5)
*Schistosoma japonicum*	1485 (12.1)
*Escherichia coli*	159 (1.3)
Returning STRING results (%)	3188 (12.0)
Returning NCBI NR results (%)	12408 (46.8)
Gene Ontology (%)	5846 (22.0)
Number of biological process terms (level 2)	24
Cellular component	20
Molecular function	14
Returning a KOBAS result (%)	5657 (21.3)
Number of predicted biological pathways	306

### Annotation of the transcriptome

Proteins predicted from *Eg*PSCs transcriptome were categorized using Blast2Go [61]. A total of 5,846 were assigned at least one GO term involved in 56 GO assignments. The predominant terms for ‘biological process’ were ‘cellular process’ and ‘metabolic process’ (19.69% and 17.42%, respectively), for ‘cellular component’ were ‘cell part’ and ‘cell’ (21.65% and 21.65%, respectively), and for ‘molecular function’ were ‘catalytic activity’ and ‘binding’ (43.41% and 40.89%, respectively) (S3 Figure).

Of the proteins predicted for *Eg*PSCs, 5,657 proteins were assigned to 306 biological pathway terms in the KEGG database (Table S1), including ‘endocytosis’ (n = 144 molecules), ‘oocyte meiosis pathway’ (n = 120), and ‘focal adhesion pathway’ (n = 118). We obtained 25 KOG clusters (S4 Figure), with 1,590 of the identified unigenes involved in at least one cluster. The largest functional group represented ‘translation, ribosomal structure and biogenesis’ (n = 214, 13.45%), followed by proteins associated with ‘post-translational modification, protein turnover, chaperones’ (n = 206, 12.95%). We also identified a further 220 (13.84%) peptidases and proteins that were linked to metabolism in eight functional categories.

### Potential secretome database

PSCs are an important, infectious component of the larval stages of *E. granulosus* that can interact with both definitive and intermediate hosts [10]. The adaptive mechanisms that facilitate this interaction between host and parasite is of great interest to our understanding of the transmission of this widespread disease. Preliminary investigations suggest that parasites secrete certain molecules to assist in host tissue colonization [13]. We therefore focused on the components of ESPs released by PSCs and their potential roles in the physiological adaptation to their hosts and/or themselves.

Of the 26,514 unigenes identified, 19,576 were translated into proteins by ESTScan, 437 proteins were predicted to be classical secreted proteins using SignalP, while 592 were predicted to be non-classical secreted proteins according to SecretomeP. The classical and non-classical proteins were then analyzed using TargetP software for mitochondrial proteins, which resulted in the removal of 25 proteins. A further 123 transmembrane proteins were removed from the secretory protein dataset by TMHMM. In total, we obtained 881 ESPs using the four tools. A further 1,399 proteins that showed a high degree of similarity to experimentally identified secreted proteins were added by the Blast program. Thus, a total of 2,280 proteins were finally predicted as secretory proteins (Table 2).

**Table 2:** Prediction of secretory-excretory proteins (ESP) from the transcriptome of EgPSCs.

Classfication	No. of predicted proteins	Prediction tools
Unigene	26514	Newbler
Protein	19576	ESTScan-3.0.3
Classic secreted proteins	437	SignalP 4.1, Web
Non-classical secretory proteins	592	SecretomeP 1.0
Mitochondrial proteins	25	TargetP 1.1, Web
Transmembrane proteins	123	TMHMM 2.0, Web
Homologues of experimentally verified proteins	1399	Blast-2.2.27
Total secreted proteins predicted	2280	

To validate the *in silico* secretome analysis, we compiled a list of all experimentally identified ESP sequences of *E. granulosus* from the NCBI database and from previous studies (47 nucleotides and 77 proteins) [9], [17], [20]–[22], and then blasted the putative ESP sequences with the known ESP sequences (see Table S2). Ninety-one proteins were successfully mapped to the known ES proteins, of which 18 shared 100% identity and 33 shared 95%–99% identity. In addition, most known ESPs from other parasites [62] were matched successfully to those identified in our study. More importantly, domains in ESPs of *Teladorsagia circumcincta* (including metridin-like ShK toxin, lectin, proteinase inhibitor I29, and allergen V5/Tpx-1) were also found in the ESPs of *Eg*PSC, which strengthens the concept that parasites employ universal ESPs to mediate parasite-host interplay [55]. Overall, these data suggest that the ESPs of *Eg*PSCs identified in this study were reliable.

To date, there have been five proteomic studies regarding *E. granulosus* that have identified just 157 ESPs among them [9], [17], [20]–[22]. In this study, approximately 500 ESP domains were found, including known proteins (Table S3), a result that significantly expands the known ES components of *Eg*PSCs. For example, WD40 repeats [63], [64], G-protein-coupled receptor (GPCR) [65] and Cadherin [66] all presented novel ESPs that were involved in parasite development-related processes. Recent studies using genome-wide and transcriptome data provide comprehensive information about the growth and development of *E. granulosus* [31], [67]. The results of this study extend this information and pave the way to a greater understanding of how PSCs utilize ESPs to survive in hosts.

### ES proteins annotation

The putative ESPs were allocated to functional categories based on InterPro domains and GO categories. Of the 2,280 proteins predicted from *Eg*PSC, the largest functional group represented ‘binding’ (n = 201, GO: 0005488), followed by ‘catalytic activity’ (n = 196, GO: 0003824) for ‘molecular function’, ‘metabolic process’ (n = 190, GO: 0008152) and ‘cellular process’ (n = 181, GO: 0009987) for ‘biological process’, and ‘cell part’ (n = 200, GO: 0044464) and ‘cell’ (n = 200, GO: 0005623) for ‘cellular component’ (S5 Figure).

The pathway enrichment analysis for identified ESPs was performed using KOBAS v2.0 software and more than 400 pathways were identified, of which 33 were statistically significant (Table 3). The term for ‘Huntington disease’ represented the most significant group (39, corrected *p*<0.0001), followed by Phagosome (37, *p*<0.0001), Protein folding (22, *p*<0.0001) and Chaperonin-mediated protein folding (16, *p*<0.0001).

**Table 3:** Pathway enrichment analysis of 1406 ESPs in the EgPSCs transcriptomes.

Category	Term^a^	Pathway database^b^	Pathway Id^c^	Sample number^d^	Background number^e^	P-Value^f^	Corrected P-value^f^
**Carbohydrate metabolism**							
Pentose phosphate pathway		KEGG	cel00030	13	18	9.41E-10	4.21E-08
Glycolysis/Gluconeogenesis		KEGG	cel00010	19	40	2.62E-08	8.80E-07
Gluconeogenesis		Reactome	—	12	19	4.87E-08	1.51E-06
Glycolysis		Reactome	—	7	8	9.66E-08	2.60E-06
Starch and sucrose metabolism		KEGG	cel00500	10	26	0.000253	0.004432
Fructose and mannose metabolism		KEGG	cel00051	8	23	0.001801	0.0226766
Amino sugar and nucleotide sugar metabolism		KEGG	cel00520	10	32	0.001974	0.0241093
Glucose metabolism		Reactome	—	18	30	2.63E-10	1.32E-08
Carbon metabolism		KEGG	cel01200	24	78	1.50E-05	0.0003552
Biosynthesis of amino acids		KEGG	cel01230	17	65	0.001585	0.0212919
**Signal transduction**							
Heterotrimeric G-protein signaling pathway		PANTHER	P00026	7	8	9.66E-08	2.60E-06
Calcium signaling pathway		KEGG	cel04020	13	37	0.000157	0.0030182
IFN-alpha/beta pathways		Reactome	—	3	5	0.001394	0.0193684
TGF-beta receptor signaling		Reactome	—	3	6	0.003739	0.0367554
Apoptosis signaling pathway		PANTHER	P00006	7	18	0.00122	0.0182041
**Proteins metabolism**							
Protein folding		Reactome	—	22	23	6.32E-21	8.49E-19
Metabolism of proteins		Reactome	—	64	293	1.75E-05	0.0003918
Mitochondrial protein import		Reactome	—	9	22	0.000238	0.0043566
Chaperonin-mediated protein folding		Reactome	—	16	19	1.57E-13	1.59E-11
Post-chaperonin tubulin folding pathway		Reactome	—	8	9	1.28E-08	5.16E-07
Activation of chaperones by ATF6-alpha		Reactome	—	5	7	3.41E-05	0.000723
Calnexin/calreticulin cycle		Reactome	—	5	12	0.002474	0.0293186
**Gene expression**							
MicroRNA (miRNA) biogenesis		Reactome	—	6	16	0.002776	0.0310737
**Genetic information processing**							
Spliceosome		KEGG	cel03040	25	103	0.000786	0.0121906
**Transport and catabolism**							
Phagosome		KEGG	cel04145	37	55	2.53E-21	5.10E-19
**Disease pathway**							
Huntington disease		PANTHER	P00029	39	41	2.31E-34	9.29E-32
Parkinson disease		PANTHER	P00049	15	31	3.76E-07	9.46E-06
**Others**							
Cytoskeletal regulation by Rho GTPase		PANTHER	P00016	15	18	1.07E-12	7.16E-11
CCT/TriC		Reactome	—	15	18	1.07E-12	7.16E-11
mRNA splicing - minor pathway		Reactome	—	13	39	0.0003	0.0050358
N-glycan trimming in ER and CNX/CRT		Reactome	—	6	13	0.000599	0.0096585
Adenine and hypoxanthine salvage pathway		PANTHER	P02723	3	6	0.003739	0.0367554

a KEGG enrichment analysis was performed by KOBAS 2.0 (http://kobas.cbi.pku.edu.cn/home.do).

*Caenorhaditis elegans* pathways were used as a reference. The ESP corresponding to each pathway can be found in Table S9.

b Pathway databases mapped by KOBAS including KEGG pathway: http://www.genome.jp/kegg/pathway.htm1; reactome: http://www.reactome.org/ReactomeGWT/entrypoint.htm1; PANTHER: http://www.patherdb.org/.

c Pathway identified in specific database.

“-” means not given.

d The number of input proteins mapped to the particular pathway.

e The number of identified proteins mapped to the particular pathway.

f Only significant results (*p*<0.05) were shown.

The statistical method was a hypergeometric test, whereas the FDR correction method was from Benjamini and Hochberg (1995).

Of the 2,280 putative ESPs, only 1,406 were mapped to known functions (Table S3). These proteins included not only many common and abundant ‘house-keeping proteins’ (e.g., ribosome proteins, cytochrome subunit proteins, and enzymes involved in carbohydrate and protein metabolism), but also some rare but interesting proteins (e.g., putative receptor and antigenic proteins). This highlights the important roles of ESPs in parasite survival and development within hostile host environments. Below, we characterize these potential ESPs in greater detail.

### Metabolism of carbohydrates for parasite energy and nutrition

The interaction of pathogens with mammalian hosts leads to a variety of physiology responses that drive the adaptation of the interacting partners to their new environments and conditions [19]. The ESPs released by parasites might be important actors in this process of adaptation, because they are involved in the metabolism of carbohydrates [68]. We identified a total of 122 domains (summarized in Table S4), of which, 32 proteins were identified to have a higher level of expression in the parasite (Table 4).

**Table 4:** The potential functional proteins with a high abundance in the ESPs from EgPSCs transcriptome.

GI Number	Description	Species^a^	Function^b^
Proteases			
116242320	Lysosomal pro-X carboxypeptidase	*C. sinensis*	
111036376	Cathepsin L-like proteinase	*E. multilocularis*	A
498980202	Lysosome membrane protein 2-like isoform X1	*M. zebra*	
226478810	Cytochrome c-type heme lyase	*S. japonicum*	
Protease inhibitor			
223037336	Kunitz protein 8	*E. granulosus*	
Structural			
124783098	Ribosomal protein S18	*T. asiatica*	
56753617	Ribosomal protein L21	*S. japonicum*	
226483022	Putative small subunit ribosomal protein S27Ae	*S. japonicum*	
256074063	60S ribosomal protein L9	*S. mansoni*	
392495090	Ribosomal protein S13	*S.erinaceieuropaei*	
421975923	60S ribosomal protein L7	*S.erinaceieuropaei*	
29841212	Putative ribosomal protein L27A protein	*S. japonicum*	
60692924	Ribosomal protein	*S. japonicum*	
421975956	Putative ribosomal protein S25	*S.erinaceieuropaei*	
358340304	U1 small nuclear ribonucleoprotein A	*C. sinensis*	
358332789	Ribosomal RNA-processing protein 9	*C. sinensis*	
256078860	U3 small nucleolar ribonucleoprotein protein imp4	*S. mansoni*	
55976640	Actin-1/4 actin	*T. solium*	
207298859	Beta-actin	*A.transmontanus*	
543766	Actin-1	*E. granulosus*	
133721998	Actin	*G. viridula*	
29337144	Tubulin beta-2 chain	*E. multilocularis*	
29337143	Tubulin beta-3 chain	*E. multilocularis*	
29337145	Tubulin beta-1 chain	*E. multilocularis*	
410897689	Tubulin alpha-1C chain-like	*T. rubripes*	
311992220	Tropomyosin 2 high molecular weight isoform	*M. corti*	A
29337029	Tropomyosin	*E. multilocularis*	A
168071448	Tropomyosin B	*E. granulosus*	A
256086965	Myosin heavy chain	*S. mansoni*	A
547974	Paramyosin	*E. granulosus*	A
432897369	Dynein light chain 2, cytoplasmic-like	*O. latipes*	A
171473974	Dynein light chain LC6	*S. japonicum*	A
405970739	Dynein light chain 2, cytoplasmic	*C. gigas*	A
68071557	Dynein light chain 1	*P. berghei*	A
29467010	Dynein light chain	*E. multilocularis*	A
226487996	Nucleolar protein 5	*S. japonicum*	
226487430	Myophilin	*S. japonicum*	A
29336625	Myophilin	*E. granulosus*	A
256086246	Histone H3	*S. mansoni*	
344240017	Histone H2A type 1	*C. griseus*	
358338242	Histone H2A.V	*C. sinensis*	
405975240	Histone H2A	*C. gigas*	
358331974	PHD finger protein 7	*C. sinensis*	
Molecular chaperone			
343887008	Heat shock protein 90 alpha	*K. marmoratus*	A, D
1661112	Heat shock 70 kDa protein, partial	*M. corti*	A
29336623	Heat shock cognate 70 kDa protein	*E. granulosus*	A
124783198	Heat shock protein gp96	*T. asiatica*	
124783152	40, partial	*T. asiatica*	A
124783287	Chaperonin	*T. asiatica*	
256082744	T-complex protein 1 epsilon subunit	*S. mansoni*	
421975972	T-complex protein 1 subunit alpha	*S.erinaceieuropaei*	
349934375	T-complex protein 1 subunit zeta	*C. sinensis*	
358342604	Molecular chaperone GrpE	*C. sinensis*	
318064648	DnaJ-like protein subfamily b member 11	*I. punctatus*	
312065499	Protein disulfide isomerase	*L. loa*	
256081230	Ubiquitin-conjugating enzyme E2r	*S. mansoni*	
29841024	26S proteasome regulatory complex subunit p42A	*S. japonicum*	
226470558	Proteasome subunit beta type 4	*S. japonicum*	
56754539	20S proteasome subunit alpha 8	*S. japonicum*	
29336773	Putative growth regulator 14-3-3	*E. granulosus*	A, ST
62178030	Putative 14-3-3 protein	*E. granulosus*	A, ST
148613837	Calreticulin	*E. granulosus*	A
444792465	Calcineurin B	*E. granulosus*	A
353530026	Calcineurin B	*E. granulosus*	A
Carbohydrate metabolism			
167541050	Phosphoglycerate mutase	*C. sinensis*	
358333945	Phosphoglycerate kinase	*C. sinensis*	
262192839	Enolase	*E. granulosus*	A
62178020	Putative glucose phosphate isomerase	*E. granulosus*	A
29336626	78 kDa glucose-regulated protein,GRP-78	*E. multilocularis*	
328789193	UTP–glucose-1-phosphate uridylyltransferase isoform 1	*A. mellifera*	
6016079	Glyceraldehyde-3-phosphate dehydrogenase	*E. multilocularis*	A
338827784	Glucose-6-phosphatase	*E. granulosus*	
470364276	UDP-glucose dehydrogenase	*C. owczarzaki*	
470610058	Cyclophilin B	*T. truncatus*	A, S, M
31077167	Cyclophilin	*T. truncatus*	A, S, M
358252886	Dehydrodolichyl diphosphate synthase	*C. sinensis*	
338827788	Phosphoenolpyruvate carboxykinase	*E. granulosus*	A
358334589	Dolichyl-phosphate beta-glucosyltransferase	*C. sinensis*	
256090534	Phosphoglucomutase	*S. mansoni*	
46406288	Malate dehydrogenase	*E. granulosus*	A
29841093	Citrate synthase	*S. japonicum*	
29336561	Fructose-bisphosphate aldolase	*E. multilocularis*	A
56682906	Hypoxanthine-guanine phosphoribosyltranferase	*S. japonicum*	
256082514	Uridine cytidine kinase I	*S. mansoni*	
358336324	Sterol O-acyltransferase	*C. sinensis*	
256085769	Methyltransferase	*S. mansoni*	
170579277	Lysyl-tRNA synthetase	*B. malayi*	
256071828	Polyadenylate binding protein	*S. mansoni*	
Oxidation/reduction			
29337026	Thioredoxin peroxidase	*E. granulosus*	A
1004227	Glutathione transferase	*E. multilocularis*	A
341616326	Peroxiredoxin 3	*C. sinensis*	A
347948498	Cu2^+^/Zn2^+^ superoxide dismutase (SOD1)	*T. solium*	A, T
29337032	Thioredoxin	*E. granulosus*	A
358340540	Thioredoxin domain-containing protein 9	*C. sinensis*	A
94556988	Neuronal nitric oxide synthase protein inhibitor	*T. solium*	PI
256070830	Peroxidasin	*S. mansoni*	A
Transporters			
256080958	Multidrug resistance protein	*S. mansoni*	
85701472	Trans-Golgi network vesicle protein 23A	*M. musculus*	
226478102	Secretory carrier-associated membrane protein 2	*S. japonicum*	
124782903	Phosphatidylinositol transfer protein alpha	*T. asiatica*	
358336646	F-type H+-transporting ATPase subunit c	*C. sinensis*	
226468748	Voltage-dependent anion-selective channel protein 2	*S. japonicum*	
392495096	Sorting nexin SNX11	*S. japonicum*	
Translation			
148717323	Elongation factor 1 alpha	*E. granulosus*	A
148717331	Elongation factor 1 alpha	*E. vogeli*	A
148717335	Elongation factor 1 alpha	*E. shiquicus*	A
159138037	RNA polymerase II elongation factor	*C. sinensis*	A
358334689	Elongation factor 2	*C. sinensis*	A
Transcription			
221509352	Zinc finger (C3HC4 type) protein	*T. gondii*	
358332148	Eukaryotictranslation initiation factor, TFIIA	*C. sinensis*	
Engery conversion			
256077755	ATP synthase beta subunit	*S. mansoni*	
226478810	Putative cytochrome c-type heme lyase (CCHL)	*S. japonicum*	
RNA Processing			
358334450	ATP-dependent RNA helicase FAL1, partial	*C. sinensis*	
Cell cycle			
353230502	Mitotic phosphoprotein 44	*S. mansoni*	
Others			
5051948	Antigen B8/1	*E. granulosus*	A
7339849	Immunogenic protein Ts11	*T. solium*	A

a The full names of species can be seen in Table S8.

b Abbreviations: A, antigenic protein; D, drug gene; ST, signal transduction; S, structural; M, molecular chaperone; T, transporters; PI, protease inhibitor.

**Figure 1:**
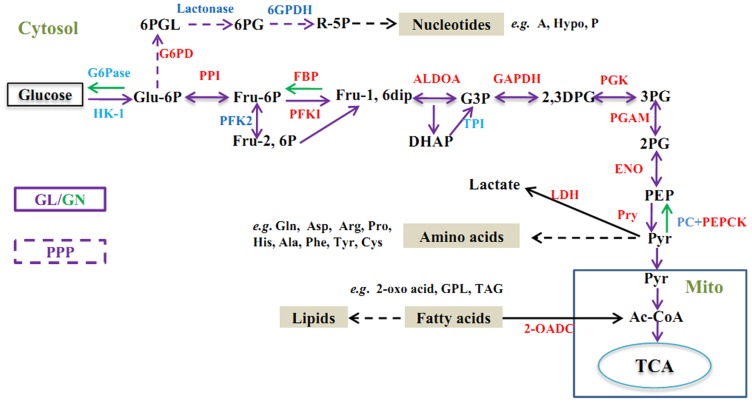
Schematic diagram showing the carbohydrate metabolic pathways involved in the ESPs of *Eg*PSCs transcriptome (Reference from Eisenreich W et al. [19] with some modifications). Glycolysis (GL, purple arrows) and gluconeogenesis (GN, grass green arrows); pentose-phosphate pathway (PPP, broken purple arrows); tricarboxylic acid cycle (TCA, blue circle) other catabolic reactions that occur in the mitochondrion and in the cytosol (black arrows). Anabolic reactions leading to amino acids, nucleotides, and lipids are indicated by broken thick black arrows. Metabolites are marked in black. Enzymes identified in our study are marked in red, while other enzymes are marked in blue. Abbreviations: HK, hexokinase; PFK, phosphofructokinase; FBP, fructose bisphosphatase; PK, pyruvate kinase; PDH, pyruvate dehydrogenase complex; PCK, PEP-carboxylase; PPI, phosphohexose isomerase; TPI, triose phosphate isomerase; PGK, phosphoglycerate kinase; GAPDH, glyceraldehydes 3-phosphate dehydrogenase; PC, pyruvate carboxylase; LDH, lactate dehydrogenase; PEPCK, phosphoenol pyruvate carboxykinase; G6PD, glucose-6-phosphate dehydrogenase; ALDOA, fructose-biphosphate aldolase; PGK, phosphoglycerate kinase; PGAM, phosphoglycerate mutase; ENO, enolase; G6Pase, glucose 6-phosphatase; G6PD, glucose-6-phosphate dehydrogenase; 6GPDH, *6*-phosphogluconatedehydrogenase. Gln, Glutanine; Asp, aspartic acid; Arg, arginine; Pro, proline; His, histidine; Ala, alanine; Tyr, tyrosine; Cys, cysteine; Ade, adenine; Hyp, hypoxanthine.

*E. granulosus* has evolved an optimal strategy to gain energy and nutrition from its host using ESPs (Fig. 1). Firstly, the parasite can regulate glycolysis (GL). We identified nine enzymes associated with GL, including the rate-limiting enzymes PFK1 and pyruvate kinase. Through GL, non-essential amino acids (e.g., glutamine, aspartic acid, arginine, proline, histidine, alanine, tyrosine and cysteine), fatty acids, adenine and hypoxanthine nucleotides, as well as pyrimidine, could be synthesized to support parasite development and growth. Alternatively, glucose and other carbohydrates could be synthesized *via* gluconeogenesis (GN) when alternative carbon sources (e.g., glucogenic amino acids, lactate, and glycerol) were available. In addition to the reversible enzymatic GL steps, several reactions are essential in the GN pathway from pyruvate via oxaloacetate to glucose: the reactions catalyzed by pyruvate carboxylase, phosphoenolpyuvate carboxykinase (PEPCK), fructose-1, 6-bisphosphatase, and glucose-6-phosphatase leading to oxaloacetate, phosphoenolpyruvate (PEP), fructose-6-phosphate, and glucose. Finally, tricarboxylic acid (TCA) enzymes, such as aconitate hydratase, succinate dehydrogenase complex, malate dehydrogenase, were identified in the TCA cycle. Other enzymes involved in carbohydrate metabolism are shown in Table 4.

Certain enzymes have been recognized to play key roles in the development of parasites. Phosphoglucose isomerase (PGI), one of glycolytic enzymes, has been found to stimulate parasite growth and the formation of novel blood vessels nearby the developing metacestode [69]. Vaccinating mice with recombinant PGI increases their resistance towards a secondary infection challenge [69]. Similarly, PEPCK is a novel egg antigen of *S. mansoni* [70] and an abundant protein in adult parasites that is related to numerous metabolic pathways (e.g., endocrine function, excretion and carbohydrate metabolism [22].

To date, only five ESPs have been identified to participate in this metabolic process [17]. The results of this study support the role of these proteins in metabolic adaptation to their hosts and, more importantly, demonstrate that many more ESPs may be used by *E. granulosus* to regulate carbohydrate metabolism. Further work is required to identify these additional ESPs and establish their functions.

### Control of parasite homeostasis

Following infection with *E. granulosus*, the intermediate host produces a significant immune response that affects the growth and development of parasites [71], [72], while the parasites initiate effective evasion mechanisms to counteract adverse host environments.

**Figure 2:**
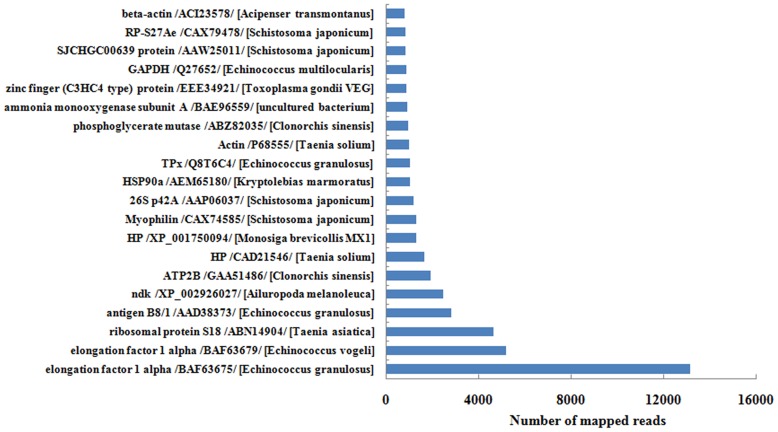
The transcription profiling of putative ESPs in *Eg*PSCs transcriptome. The 20 most abundant ESPs encoded in the transcriptome are shown. Abbreviations: RP-S27Ae, putative small subunit ribosomal protein S27Ae; GAPDH, glyceraldehydes-3-phosphate dehydrogenase; ndk, nucleoside diphosphate kinase B-like; ATP2B, Ca_2_^+^ transporting ATPase plasma membrane; HSP90α, heat shock protein alpha; 26S p42A, 26S proteasome regulatory complex subunit p42A.

In this study, we found that 36 ESP domains were molecular chaperones (Table S5), and identified a further 25 proteins that were present with high levels of abundance (Table 4), including several novel molecules (heat shock proteins, HSP90 and HSP40, universal stress protein [Usp], calreticulin, calcineurin B, GrpE in the HSP60 family and Gp96). HSP90 was the most strongly expressed of all the molecular chaperons (Fig. 2), suggesting it is one of the key molecules in mediating parasite development. This is supported by the fact that nitration of HSP90 is known to induce cell death [73], and HSP90 has been used as a drug target in protozoa intervention [74]. Previous studies have also shown that UspA and Usp8 are associated with stress resistance and growth in bacterial species [75]. ESPs might disrupt the expression of intracellular 70 protein in the host immune cells, while the parasite itself might release HSP70 to prevent damage from those same cells [76]. These molecular chaperone-like proteins may be released to regulate the stress responses that arise in the extremely harsh intestinal environments of definitive hosts (e.g., numerous highly active proteases, variable pH levels).

*E. granulosus* may secrete proteases or inhibitors to digest host proteins, or to protect itself from digestion by endogenous or host-derived proteinases. In this study, 39 proteases, including serine, aspartic, metallo- and cysteine proteinases, and five inhibitors, were inferred among the set of ESPs (see Table S6). Several of these (serine, cysteine, and the proteinase inhibitors) are likely to be important targets for parasite intervention and control [77]–[79]. However, only three proteases and two protease inhibitors were strongly expressed in the set of ESPs (Table 4). More sensitive technologies will therefore be required to identify other proteases that were expressed at lower levels of abundance.

In contrast, the action of antioxidant enzymes is a key component of parasite survival during infection. In this study, seven ESPs were identified as antioxidant enzymes, including glutathione transferase, peroxiredoxin, thioredoxin, Cu_2_^+^/Zn_2_^+^ superoxide dismutase, and neuronal nitric oxide synthase protein inhibitor. These molecules might be utilized by the parasite to detoxify the reactive oxygen species produced by the host environments [80].

### Direct regulation of host immunological responses

In previous experiments we demonstrated that following infection with *E*gPSCs the microenvironment of the murine peripheral immune system undergoes several changes. These included T cell activation and the accumulation of immunosuppressive cells, such as myeloid-derived suppressor cells (MDSC) and CD4^+^CD25^+^FoxP3^+^ T cells (Treg) [71]. Such alterations might occur *via* the action of ESPs as many ESPs have been found to redirect host immune responses [13], [17]. In this study, we found several ESPs that contribute to immune regulation following infection (Table 4). Tegument protein (Teg) is known to induce a biased Th2 cell immune response related to chronic infection [81], while 14-3-3 proteins are associated with resistance to the immune responses mediated by local cells [82]. In addition, the antigen B (AgB) family are important in immune evasion because the antigen is secreted at variable amounts [83], and have also been demonstrated to direct immature DC maturation towards a preferential Th2 immune response [15].

Notably, cysteine proteinases have been reported to inhibit Th1 immune response *via* the induction of IL-4, which is the main cytokine responsible for Th2 differentiation [84]. HSP70 has been shown to stimulate both of types of response in CHD patients [85]. Also, the intraperitoneal injection of calreticulin (CRT) significantly influences Th1/Th2 balance [86]. Hence, these proteins might be novel immunoregulatory molecules that contribute to immune evasion.

### Signaling pathways

We found that *Eg*PSC possesses many signaling pathways such as P13K-Akt, mitogen-activated protein kinase (MAPK), Wnt, calcium, HIF-1, insulin, estrogen and chemokine signaling (Table S1). However, in the putative set of ESPs, only G-protein, calcium, IFN-α/β, TGF-β receptor and apoptosis signaling pathways were dominant (Table S7), which indicated their importance in parasite-host interactions and physiological processes.

Notably, we found that G-protein-coupled receptors (GPCRs), TGF-β and insulin signaling pathways might closely associate with the development of *Eg*PSCs. For example, GPCRs can activate the G-proteins located within the cell. They work cooperatively to deliver varied signals, which in turn regulate various physiological processes [87]. However, the exact function of G-protein signaling in parasites remain unclear.

Studies have shown that TGF-β and insulin signaling pathways in *C. elegans* can trigger an ‘alternative’ developmental pathway, and can regulate and transit the environmental stresses on the first larval stage of the parasite [88], [89]. In particular, the disruption of both signaling pathways leads to arrested development in this species [90], [91]. Indeed, the TGF-β pathway is speculated to regulate developmental events in parasitic nematodes [92], as molecules involved in the TGF-β pathway have been found in several parasitic nematodes including *Brugia pahangi*, *Brugia malayi* and *Parastrongyloides trichosuri* [93]–[95]. The role of TGF-signaling in *E. granulosus* development and growth warrants further investigation. A recent study revealed that host insulin acts as a stimulant for parasite development within the host liver and that *E. multilocularis* senses the hormones of hosts through an evolutionary-conserved insulin signaling pathway, which demonstrates the importance of insulin signaling for parasite survival [96].

### Potential targets for diagnosis and vaccine development

CHD has a global distribution and causes high rates of morbidity and has a high socio-economic burden in several countries [97]. The Eg95 vaccine induces a high antibody titer in sheep and goats, which protects them against CHD [98]. However, due to antigenic variation caused by genotypic diversity [99], the common Eg95 vaccine does not bind the antibodies of all *E. granulosus* species, which limits its utility. We suggest that the ESPs of *Eg*PSCs are an excellent alternative candidate for a vaccine, as they are easy to prepare and safer for human health. More importantly, the ESPs obtained by *in vitro* culture have shown a 92.07% protection rate against a high dose of egg infection in sheep (1,000 eggs per sheep) [100].

Using *in silico* secretome analysis, we identified 44 antigenic proteins present at high abundance in our set of ESPs (Table 4). Of these, elongation factor 1 alpha, antigen B8/1, myophilin, thioredoxin peroxidase, phosphoglycerate mutase, heat shock protein 90a and actin, were the most abundant. In addition, HSP70, enolase, 14-3-3, phosphate glucose isomerase, malate dehydrogenase, glutathione S-transferase were also present at high abundance in the set of ESPs (Table S8). These abundant proteins hold enormous potential as diagnostic markers or intervention targets. Indeed, malate dehydragenase (MDH) has been tested for the immunodiagnosis of *E. granulosus*, while thiredoxin peroxidase (TPx) has been used for the immunodiagnosis of human CHD [101]. Likewise, the 14-3-3 molecule has been demonstrated to be a candidate vaccine against *E. granulosus* in mice [12], while recombinant GST protein has been used in the diagnosis of echinococcosis [102].

Proteins that are present at lower levels of abundance might also be relevant as diagnostic markers or target molecules for vaccine development. In this study, these include antigen 5 (Ag5), calreticulin, calcineurin B, thioredoxin, phosphoglucomutase, fructose-bisphosphate aldolase and gp96 (Table S8). Many of these have already shown promise for serodiagnostic purposes. For example, Ag5 is a dominant immunogenic and diagnostic antigen of the *E. granulosus* metacestode in both adults and PSCs [22]. Similarly, calcineurin B has been previously identified as a candidate for a vaccine or drug target [103]. Surprisingly, the *E. granulosus*-specific protein domain antigen B (*Eg*AgB) family, which are well known as diagnostic targets, were undetectable in this study. This result was consistent with previous observations that little or no AgB is secreted by *in vitro* cultured PSCs [17], [104]. Previous studies have demonstrated that the germinal layer, but not the PSC, contributes to the primary secretion of AgB [17]. Thus, serological examination based on the AgB antibody would not be useful in early-stage PSC infection as only minute amounts of AgB antibody are produced at that time.

There are currently just two methods for the treatment of hydatid disease: surgery and the use of benzimidazole, both of which give unsatisfactory results. Hence, novel treatment compounds are urgently needed. In this study, we have identified several secretory drug targets for echinococcosis (Table 4, Table S3), including GPCRs, threonine and tyrosine protein kinase and nuclear hormones, which have been the targets of successful new drug discoveries [65]. Insulin signaling [96], thyrotropin-releasing hormone receptor, pancreatic hormone-like or transforming growth factor-β (TFG-β) families have been linked to the larval developmental of *E. multilocularis*. Thus, interventions that utilize these molecules could also arrest parasite growth. In addition, GL enzymes could be drug targets for parasites that rely on the GL pathway for growth and development [22]. Finally, HSP90 has been used as a drug target in protozoa intervention programs [74].

### Conclusions

The larval stages of *E. granulosus* are pathogenic to human, which therefore have become the research focus of CHD. Parkinson et al. [2012] first reported genes with features that reflect physiological adaptations of different parasite stages, including PSCs, and revealed abundant long non-protein coding transcripts, upregulated fermentative pathways, candidate apomucins and a set of platyhelminth-specific gene products, which greatly increased the quality and the quantity of the molecular information regarding *E. granulosus* [67]. The most newly published genome of the parasite also uncovered several key events of the parasites, including the species-specific genes AgB family, bile salt pathways and Cavβ1 gene variation associated with praziquantel sensitivity [31]. Those studies have provided a molecular understanding of the growth and development of *E. granulosus*. In this study, we focused on the transcriptome of PSCs, which is the only infective component of the larval stages. We present novel and urgently needed information regarding the components of ESPs released by PSCs and their potential roles in the metabolic adaptation of parasites to their hosts. We suggest that intracellular ESPs are essential to the metabolism of carbohydrates within their hosts and that various molecular chaperones with a high level of expression may play a role in resisting harsh host environments. We also reveal a set of antigenic ESPs that show promise as candidates for vaccine development or in the development of serodiagnostic markers. Such findings will encourage more novel strategies for the treatment and control of CHD.

Although the coverage of the transcriptome data in this study was not deep as the genome-wide study [31], [67], these findings are novel and hold importance for understanding the mechanisms of parasite metabolic adaptations within their hosts. Overall, this study adds supplementary knowledge regarding the genomics of *E. granulosus*, and deepens our understanding of host-parasite interactions.

## Supporting Information

S1 Figure **Genotype identification of** ***E. granulosus*** **.** (A) PCR amplification. M, DNA maker; Cytb, 601 bp; Cox1, 885 bp. (B) Sequence alignment of the cytochrome b (cytb) gene. Bases that differed are marked with red boxes.
(TIF)

S2 Figure **Length distribution of singletons and isotigs of the** ***Eg*** **PSCs transcriptome.**
(TIF)

S3 Figure **Gene ontology (GO) analysis of the** ***Eg*** **PSCs transcriptome.** BLASTP against SwissProt and GO mapping of identified proteins (performed with BLAST2GO) [61].
(TIF)

S4 Figure **Distribution of the KOG functional categories of the proteins identified from the** ***Eg*** **PSCs transcriptome.** Percentages and numbers of proteins in each functional category are indicated in the sectors of the circle. KOG functional categories: (A) RNA processing and modification; (B) Chromatin structure and dynamics; (C) Energy production and conversion; (D) Cell cycle control, cell division, chromosome partitioning; (E) Amino acid transport and metabolism; (F) Nucleotide transport and metabolism; (G) Carbohydrate transport and metabolism; (H) Coenzyme transport and metabolism; (I) Lipid transport and metabolism; (J) Translation, ribosomal structure and biogenesis; (K) Transcription; (L) Replication, recombination and repair; (M) Cell wall/membrane/envelope biogenesis; (N) Cell motility; (O) Posttranslational modification, protein turnover, chaperones; (P) Inorganic ion transport and metabolism; (Q) Secondary metabolites biosynthesis, transport and catabolism; (R) General function prediction only; (S) Function unknown; (T) Signal transduction mechanisms; (U) Intracellular trafficking, secretion, and vesicular transport; (V) Defense mechanisms; (W) Extracellular structures; (Y) Nuclear structure; (Z) Cytoskeleton. The number of proteins in the graphic might exceed the total of predicted ESP because some were grouped in more than one functional category.
(TIF)

S5 Figure **Gene ontology (GO) analysis of the identified ESPs from the** ***Eg*** **PSCs transcriptome.** The figure shows the number of mapped proteins identified in this study as a function of all the available GO terms of level 2 for (A) biological process, (B) cellular component, and (C) molecular function.
(TIF)

S1 Table **KEGG pathway analysis of the** ***Eg*** **PSCs transcriptome sequences.**
(XLSX)

S2 Table **Validation evaluation of the predicted ESPs from the** ***Eg*** **PSCs transcriptome.**
(XLS)

S3 Table **Overview of the predicted ESPs from the** ***Eg*** **PSCs transcriptome.** ESPs were conceptually translated and inferred from the coding domains of transcriptomic sequences. Domain analysis of ESPs was then carried out using InterProScan.
(XLS)

S4 Table **Domains associated with carbohydrate metabolism in the ESP.**
(XLSX)

S5 Table **Domains related to post-translational modification, protein turnover, and chaperones in the ESPs.**
(XLSX)

S6 Table **Domains of the proteases and protease inhibitors in the ESPs.**
(XLSX)

S7 Table **Overview of the KEGG pathways involved in the predicted ESPs.**
(XLSX)

S8 Table **The most abundant transcripts in the ESPs of the** ***Eg*** **PSCs based on RPKM (reads per kilobase per million reads).**
(XLSX)

S9 Table **The proteins that were significantly enriched in the KEGG pathways of the predicted ESPs.**
(XLSX)
