# Supplementary figures and images for: Transcriptome Profiles of the Protoscoleces of Echinococcus granulosus Reveal that Excretory-Secretory Products Are Essential to Metabolic Adaptation
Source: PLoS Negl Trop Dis. 2014 Dec 11;8(12):e3392. doi: 10.1371/journal.pntd.0003392 (PMC4263413; doi:10.1371/journal.pntd.0003392)

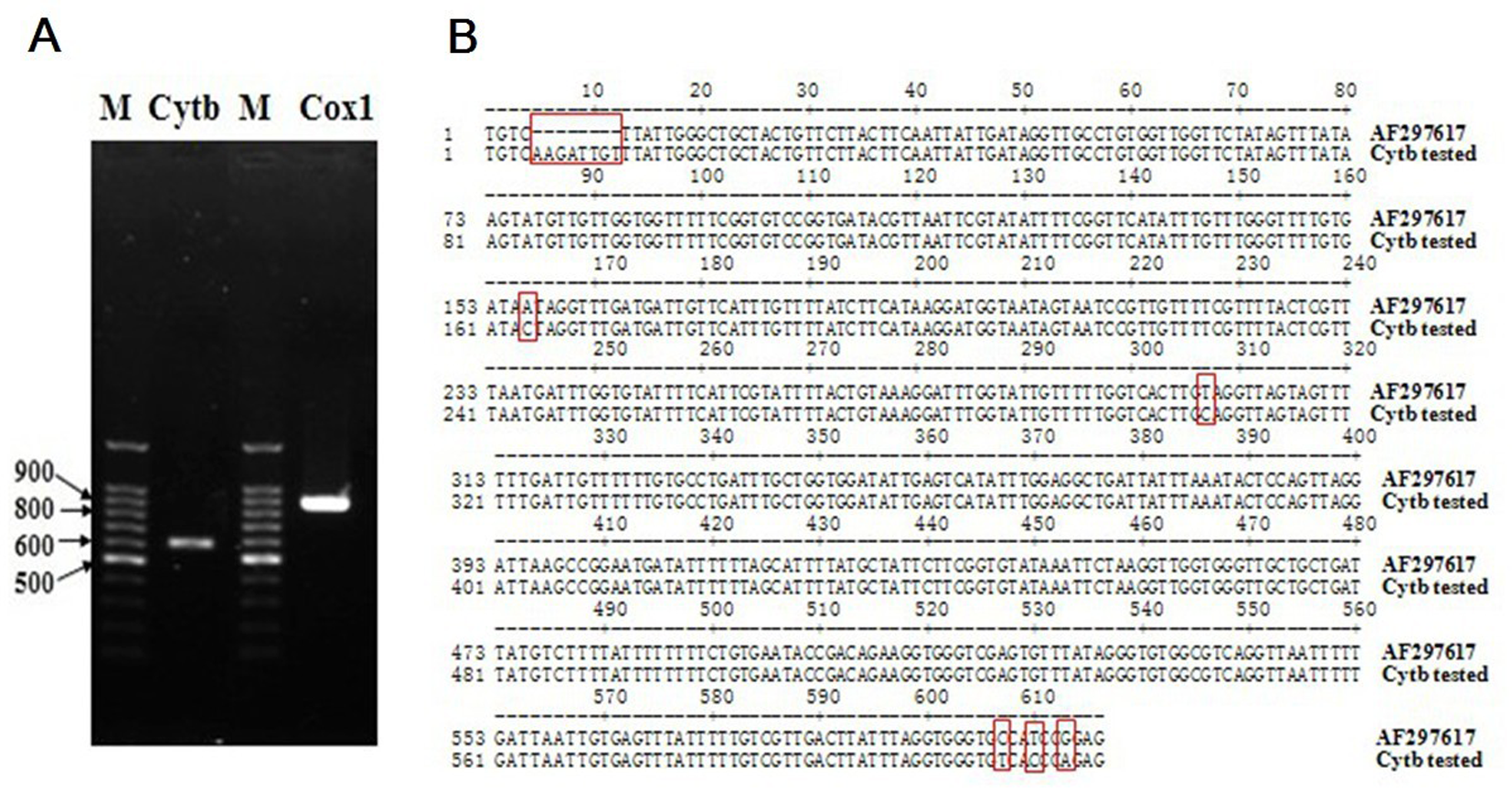

Supplement: S1 Figure — Genotype identification of E. granulosus . (A) PCR amplification. M, DNA maker; Cytb, 601 bp; Cox1, 885 bp. (B) Sequence alignment of the cytochrome b (cytb) gene. Bases that differed are marked with red boxes. (TIF) [file pntd.0003392.s001.tif]

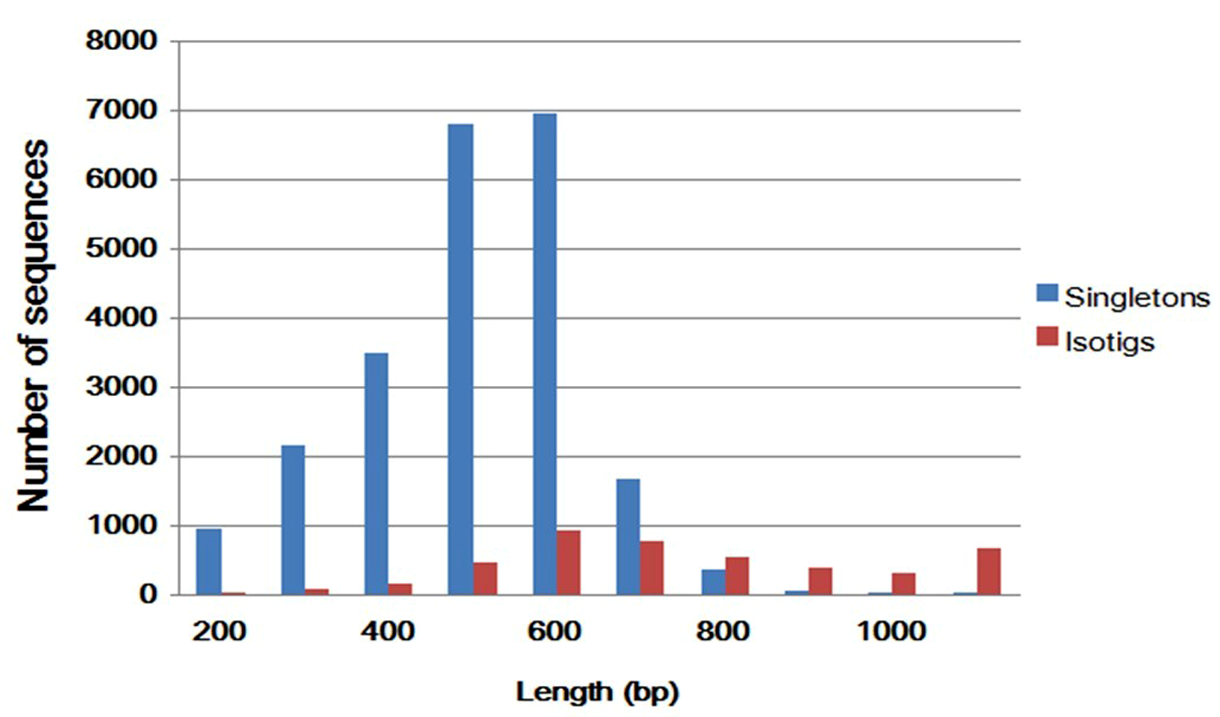

Supplement: S2 Figure — Length distribution of singletons and isotigs of the Eg PSCs transcriptome. (TIF) [file pntd.0003392.s002.tif]

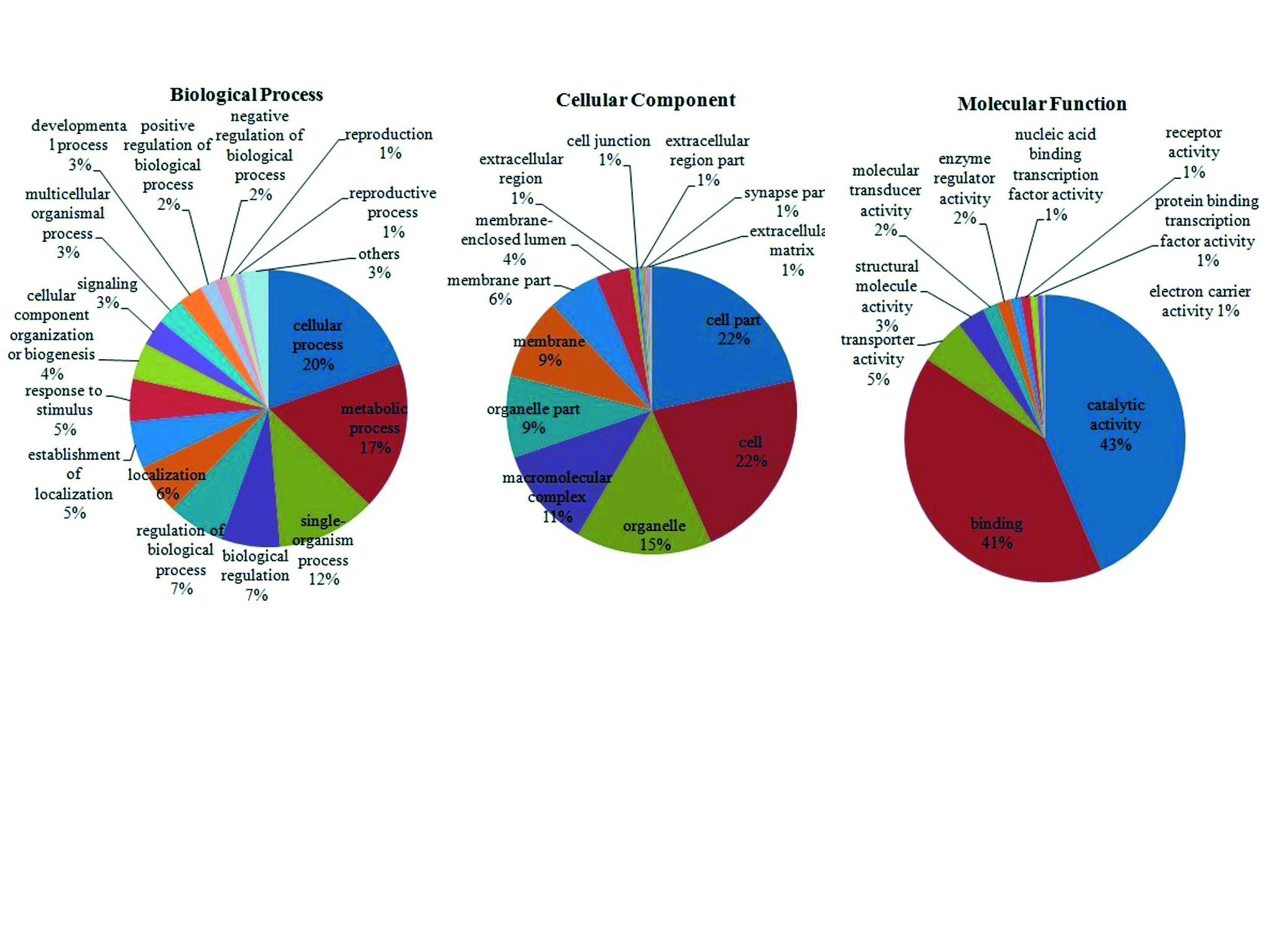

Supplement: S3 Figure — Gene ontology (GO) analysis of the Eg PSCs transcriptome. BLASTP against SwissProt and GO mapping of identified proteins (performed with BLAST2GO) [61]. (TIF) [file pntd.0003392.s003.tif]

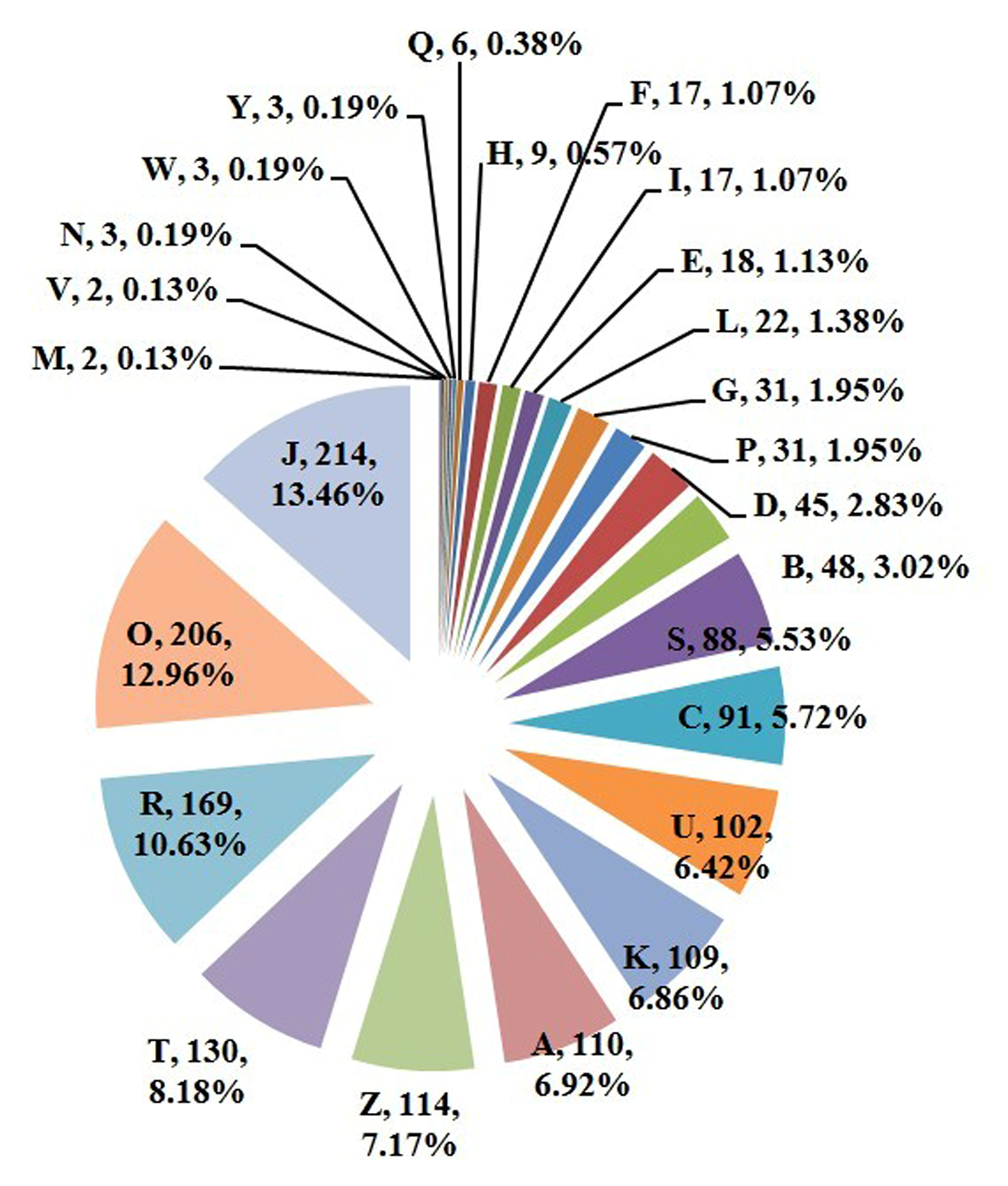

Supplement: S4 Figure — Distribution of the KOG functional categories of the proteins identified from the Eg PSCs transcriptome. Percentages and numbers of proteins in each functional category are indicated in the sectors of the circle. KOG functional categories: (A) RNA processing and modification; (B) Chromatin structure and dynamics; (C) Energy production and conversion; (D) Cell cycle control, cell division, chromosome partitioning; (E) Amino acid transport and metabolism; (F) Nucleotide transport and metabolism; (G) Carbohydrate transport and metabolism; (H) Coenzyme transport and metabolism; (I) Lipid transport and metabolism; (J) Translation, ribosomal structure and biogenesis; (K) Transcription; (L) Replication, recombination and repair; (M) Cell wall/membrane/envelope biogenesis; (N) Cell motility; (O) Posttranslational modification, protein turnover, chaperones; (P) Inorganic ion transport and metabolism; (Q) Secondary metabolites biosynthesis, transport and catabolism; (R) General function prediction only; (S) Function unknown; (T) Signal transduction mechanisms; (U) Intracellular trafficking, secretion, and vesicular transport; (V) Defense mechanisms; (W) Extracellular structures; (Y) Nuclear structure; (Z) Cytoskeleton. The number of proteins in the graphic might exceed the total of predicted ESP because some were grouped in more than one functional category. (TIF) [file pntd.0003392.s004.tif]

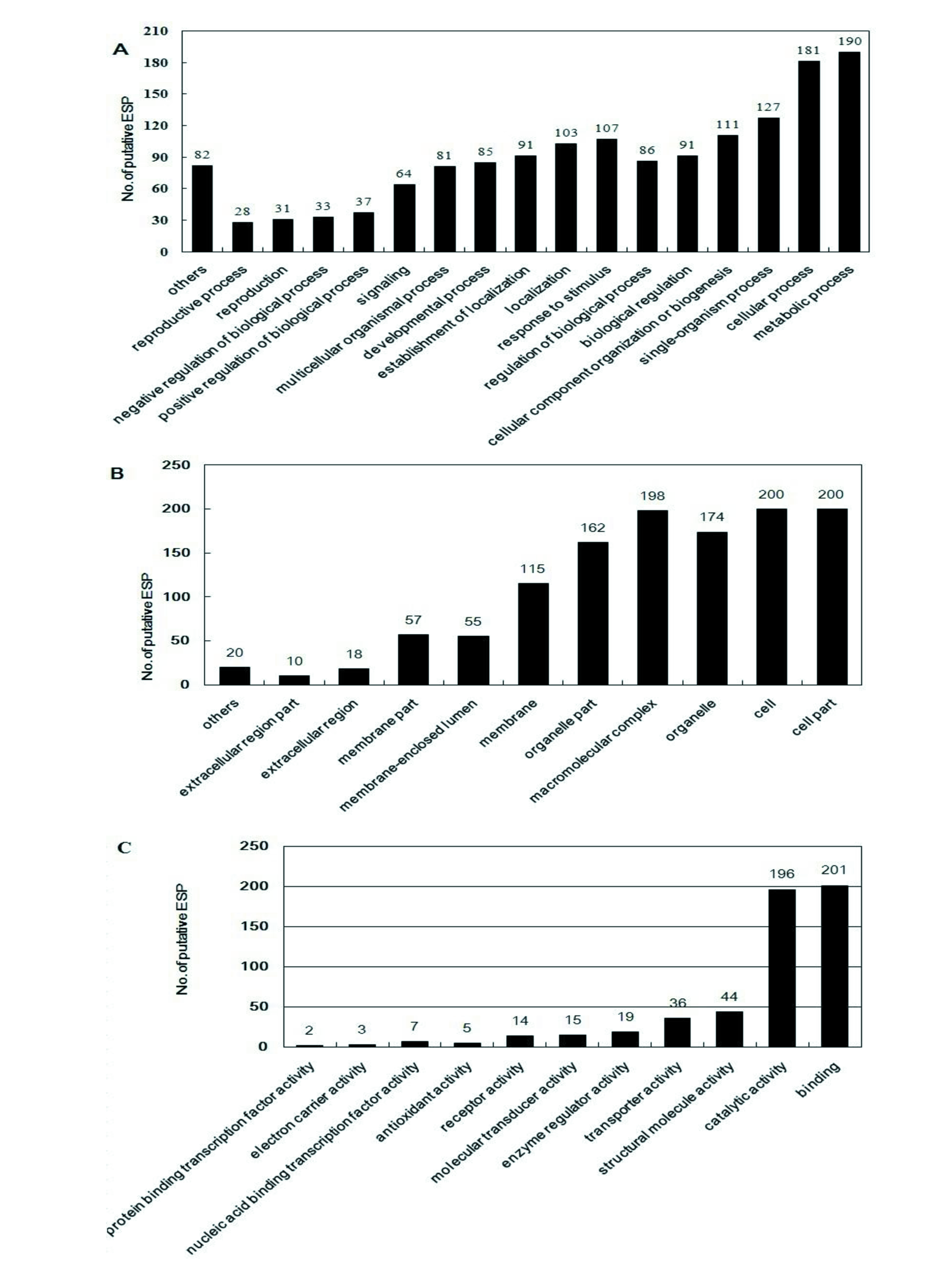

Supplement: S5 Figure — Gene ontology (GO) analysis of the identified ESPs from the Eg PSCs transcriptome. The figure shows the number of mapped proteins identified in this study as a function of all the available GO terms of level 2 for (A) biological process, (B) cellular component, and (C) molecular function. (TIF) [file pntd.0003392.s005.tif]
